# Proprioceptive training on the recovery of total knee arthroplasty patients: A meta-analysis protocol of randomized controlled trial

**DOI:** 10.1097/MD.0000000000023757

**Published:** 2020-12-18

**Authors:** Jia-qi Wu, Hong-wei Bao, Lin-bo Mao, Ling-feng Liu, Yong-mei Li, Jing-zhao Hou, Can-hua Wu, Yue-jiang Zhou, Zhao Wang, Yan-xiao Cheng, Jian Wu

**Affiliations:** a Rehabilitation Department; b Department of Orthopedics, Jingjiang People's Hospital; c Institute Office, Jingjiang People's Hospital, Jingjiang, Taizhou City, Jiangsu Province, China.

**Keywords:** meta-analysis, proprioceptive training, protocol, total knee arthroplasty

## Abstract

**Background:**

Total knee arthroplasty is a common surgery for end-stage of knee osteoarthritis. Proprioceptive training has become an important part in athletes training programmes in different sports. However, the effects of proprioceptive training on the recovery of total knee arthroplasty were unknown. This meta-analysis, with its comprehensive and rigorous methodology, will provide better insight into this problem.

**Methods and analysis:**

Electronic databases including PubMed, EMBASE, Web of Science, China National Knowledge Infrastructure (CNKI) database, Wanfang Database and Chinese Biomedical Literature Database (CBM) were searched from its inception to October 21, 2020. We only included proprioceptive training vs placebo in patients after total knee arthroplasty and pooled results were summarized by STATA 12.0 software. Two researchers independently selected the study and assessed the quality of the included studies. The heterogeneity was measured by *I*^2^ tests (*I*^2^ < 50 indicates little heterogeneity, *I*^2^ ≥ 50 indicates high heterogeneity). Publication bias was ruled out by funnel plot and statistically assessed by Beggs test (*P* > .05 as no publication bias).

**Results:**

Results will be published in relevant peer-reviewed journals.

**Conclusion:**

Our study aims to systematically present the clinical effects of proprioceptive training after total knee arthroplasty patients, which will be provide clinical guidance for total knee arthroplasty patients.

## 1 Introduction

Total knee arthroplasty is a common surgical procedure for a painful end-stage knee osteoarthritis (OA).^[1–3]^ It affects millions of people worldwide, with knee OA being the most common in terms of prevalence (6% of adults > 30 years of age).^[4,5]^ Total knee arthroplasty costs exceeded US$1087.43 million.^[6]^ These numbers are expected to rise further due to increasing proportion of aging population and obese population.^[7]^ Total knee arthroplasty is an effective treatment that helps to reduce knee pain and can increase physical performance in patients with severe OA.^[8]^ In general, the effectiveness of total knee arthroplasty has been unsatisfactory, especially for the postoperative rehabilitation.

Proprioceptive training programmes using special training tools, such as wobble boards or soft balance-pads, have become an important element in various sports.^[9–11]^ Proprioceptive training is known to lead to an economisation of movements and to help perform energy-saving movement patterns.^[12]^ Therefore, it is necessary to explore additional effective rehabilitation protocols after total knee arthroplasty.

In this meta-analysis, we systematically reviewed relevant published articles about proprioceptive training after total knee arthroplasty to analyses the effectiveness of proprioceptive training after total knee arthroplasty.

## 2 Methods and analysis

### 2.1 Study registration

The Preferred Reporting Items for Systematic Reviews and Meta-Analysis Statement (PRISMA) was applied for guiding this systematic review and meta-analysis.^[13]^ This meta-analysis was registered in the Registry of Systematic Review/Meta-Analysis (https://www.researchregistry.com/browse-the-registry#registryofsystematicreviewsmeta-analyses/, No. reviewregistry1023). And this study protocol was funded through a protocol registry. This study receives ethics approval from Jingjiang People's Hospital and founded by Minsheng Science and Technology Project of Suzhou (SS201814).

### 2.2 Inclusion and exclusion criteria

The details regarding the inclusion criteria are listed as follows:

1. Patients: Diagnosis of knee OA was defined using the American College of Rheumatology (ACR) criteria of classification of OA of the knee;
2. Intervention: proprioceptive training;
3. Control: placebo;
4. Outcomes: self-reported functionality, quality of life, Biodex Balance System and knee function; and
5. Study design: randomized controlled trials.

The study selection process was carried out by 2 reviewers, and any conflict between 2 reviewers was resolved by an additional reviewer evaluation the full-text of article.

### 2.3 Study search

We systematically searched the PubMed, EMBASE, Web of Science, China National Knowledge Infrastructure (CNKI) database, Wanfang Database and Chinese Biomedical Literature Database (CBM) to identify included trials through October 21, 2020, and using the following core terms: (“total knee arthroplasty” OR “total knee replacement” OR “TKA” OR “TKR”) AND (“proprioceptive training”). The studies have already completed but not yet published were searched from the website http://clinicaltrials.gov/ (US NIH) and the Meta Register of Controlled Trials. We also reviewed the reference lists of identified studies to identify any new RCT met the inclusion criteria.

### 2.4 Study selection

**Figure 1:**
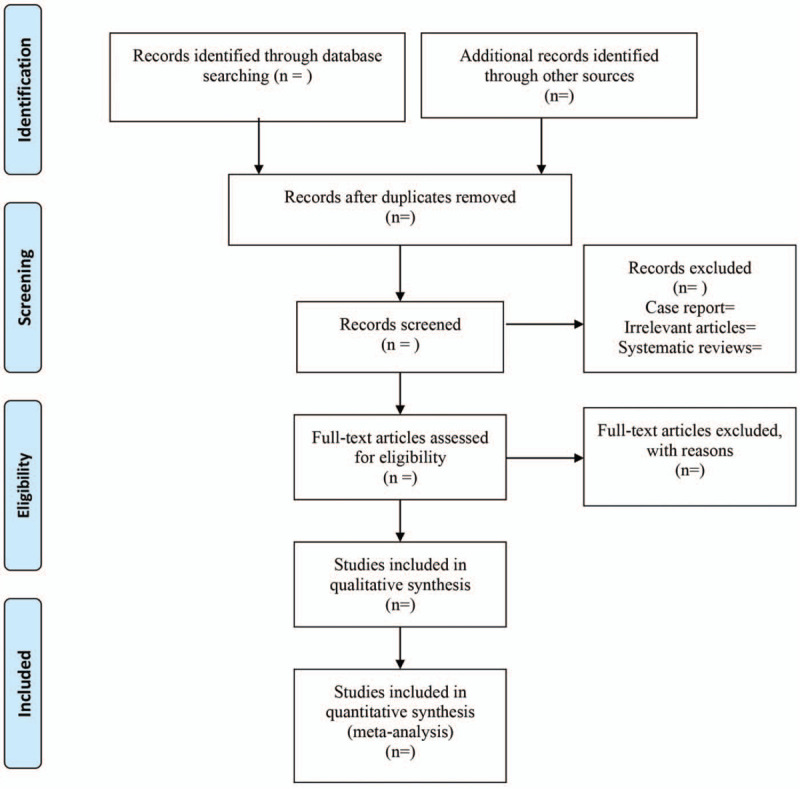
The flow diagram of procedure to select studies.

EndNote X9 (Thomson Reuters, Toronto, Ontario, Canada) was used for literature managing and records literature selection. Study selection was conducted independently by 2 reviewers (Jia-qi Wu and Hong-wei Bao) and discrepant results were resolved by discussion until a unanimous decision was reached. The study flow chart is presented in Figure 1.

### 2.5 Data extraction

The following information were abstracted by 2 reviewers independently: first authors name, publication year, country, sample size, percentage male, mean age, type of operation, anesthesia, intervention, control, and investigated outcomes, any disagreement between reviewers was settled by discussion until a consensus.

### 2.6 Risk of bias assessment

Two researchers (Yan-xiao Cheng and Jian Wu) independently assessed the quality of the included trials based on Cochrane risk of bias assessment tool. This tool mainly including 7 items: random sequence generation, allocation concealment, blinding of participants and personnel, blinding of outcome assessment, incomplete outcome data, selective reporting and other bias.

### 2.7 Data analysis

The treatment effectiveness between proprioceptive training and placebo for self-reported functionality, and knee function were assigned as continuous data, and weighted mean difference (WMD) with its 95% confidence interval (CI) was calculated in each trial. Moreover, the quality of life, Biodex Balance System were assigned as categories data, and relative risk (RR) with 95%CI in each trial before data pooling. The pooled analyses for investigated outcomes were conducted applied by the random-effects model.^[14,15]^ Heterogeneity across included studies were assessed by *I*^2^ and Q statistic, and significant heterogeneity was defined as *I*^2^ > 50% or *P* < .10.^[16,17]^ Subgroup analyses were also conducted on the basis of time endpoints and the type of operation. Publication biases for investigated outcomes were assessed by the Egger and Begg tests.^[18,19]^ The inspection level for pooled results are 2-sided, and *P* < .05 was regarded as statistically significant. The STATA software (Version 14.0; StataCorp, Texas, United States of America) was applied to conduct all statistical analyses in this study.

## 3 Discussion

The aim of this meta-analysis was to summarize the existing evidences about proprioceptive training after total knee arthroplasty. This study has some highlights. First, this is the first systematic review and meta-analysis about the effectiveness of proprioceptive training after total knee arthroplasty. In addition, we systematically searched the both English and Chinese databases to comprehensively selected the published papers. These methods demonstrate the reliability of our study. Moreover, we performed subgroup analysis and sensitivity analysis to increase the reliability of our meta-analysis. Consistency between reviewers were identified by kappa value. Finally, we could provide evidence for clinical guidance.

## Acknowledgment

We would thank for the Registry of Systematic Review/Meta-Analysis platform for registry for this meta-analysis.

## Author contributions

**Conceptualization:** Jia-qi Wu, Jing-zhao Hou.

**Data curation:** Jing-zhao Hou.

**Formal analysis:** Jia-qi Wu.

**Investigation:** Jia-qi Wu, Yong-mei Li, Yue-jiang Zhou.

**Methodology:** Jia-qi Wu, Yong-mei Li.

**Resources:** Lin-bo Mao.

**Software:** Can-hua Wu, Yan-xiao Cheng, Jian Wu.

**Supervision:** Yue-jiang Zhou, Jian Wu.

**Validation:** Hong-wei Bao, Jian Wu.

**Visualization:** Hong-wei Bao.

**Writing – original draft:** Ling-feng Liu, Zhao Wang.

**Writing – review & editing:** Hong-wei Bao, Ling-feng Liu, Zhao Wang.
